# Gut‐Brain Axis in Obesity: How Dietary Patterns Influence Psychological Well‐Being and Metabolic Health?

**DOI:** 10.1002/fsn3.70689

**Published:** 2025-07-24

**Authors:** Faiyaz Ahmed, Muhammad Tayyab Arshad, Sammra Maqsood, Ali Ikram, Kodjo Théodore Gnedeka

**Affiliations:** ^1^ Department of Basic Health Sciences, College of Applied Medical Sciences Qassim University Buraydah Saudi Arabia; ^2^ Functional Food and Nutrition Program, Faculty of Agro‐Industry Prince of Songkla University Songkhla Thailand; ^3^ University Institute of Food Science and Technology The University of Lahore Lahore Pakistan; ^4^ National Institute of Food Science and Technology University of Agriculture Faisalabad Faisalabad Pakistan; ^5^ Togo Laboratory: Applied Agricultural Economics Research Team (ERE2A) University of Lomé Lome Togo

**Keywords:** gut microbiota, insulin resistance, obesity, probiotics

## Abstract

Combining nutritional, microbial, and psychological factors, the gut‐brain axis plays a pivotal role in obesity. In distinction to Western diets which are heavy in processed foods and disturb gut microbiota leading to inflammation and metabolic dysfunction while Mediterranean diets which are rich in fiber and probiotics improve microbial diversity and psychological health. Emotional consumption, aggravated by psychological states such as sadness and stress, is a prominent driver of the obesity epidemic. Nutritional interventions such as prebiotics and fiber are needed to restore metabolic and emotional balance since dysbiosis in the gut has been linked with insulin resistance and type 2 diabetes. Recent findings demonstrate that treatment could be optimized through tailored dietary intervention depending on microbial patterns. One potential all‐encompassing strategy for the treatment of obesity is an integrative strategy that combines dietary modification with psychological counseling and pharmacologic therapies. Diet, gut and mental health are inversely correlated with each other, and this review summarizes the current evidence on the gut‐brain axis in obesity. To treat the multi‐factorial etiology of obesity, future research should explore drugs that act on the microbiome as well as multi‐disciplinary strategies.

## Graphical abstract

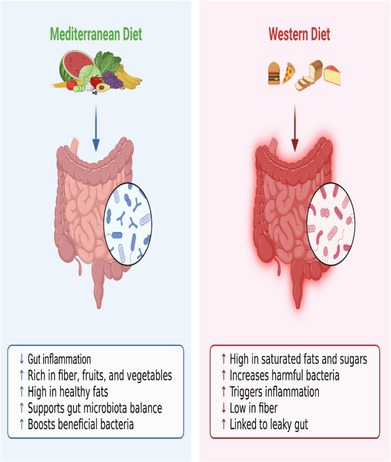

## 1 Introduction

Obesity is now a leading public health issue worldwide (Blüher 2019). Globally, individuals of all economic statuses and ages are increasingly getting obese, a condition characterized by adverse levels of excess fat storage (James 2018). More than 650 million individuals were obese in 2016, and the incidence of obesity has almost doubled since 1975, as reported by Blüher (2019). This chronic health condition is a dominant cause of global morbidity and mortality because of the high rate of comorbidities, such as type 2 diabetes (T2D), cardiovascular disease, cancer, and psychiatric diseases (Jaacks et al. 2019).

Obesity is increasingly becoming prevalent in low‐ and middle‐income countries that are undergoing rapid dietary transitions and urbanization, as per global trends (Jaacks et al. 2019). The “obesity transition” refers to a shift from undernutrition to overnutrition due to excessive consumption of calorie‐dense foods with low nutrient value and physical inactivity. Systemic gaps in the prevention and management of obesity exist, according to Friedrich (2017), since the global burden of obesity continues to rise even with increased public awareness and advances in medicine. Adding to its complexity of management are the socio‐economic aspects of obesity (Huang et al. 2024). Obesity is gender and income level‐dependent, as discussed by Ameye and Swinnen (2019). The poorer parts of society tend to be more affected by the disease; subsequently, they have limited access to medical services and more healthy food. On the contrary, Seidell and Halberstadt (2015) debate that fighting obesity demands an integrated policy taking into account economic, cultural, and environmental features in addition to biological aspects.

Acknowledgments to the latest advances in nutrition science, microbiology, and neurology, one of the key concepts to grasp obesity has appeared: the gut‐brain axis (GBA) (Zhu et al. 2022). The GBA is well‐defined by Berthoud et al. (2021) as the neuronal, endocrine, and immunological networks of paths participating in bidirectional communication among the CNS and gastrointestinal tract. The regulation of hunger, energy homeostasis, mood, and behavior states that are intensely associated with obesity's pathophysiology is attained by this axis. The gut microbiota, which is an assemblage of polymicrobial microbes found in the gastrointestinal system, is particularly concerned in the GBA. The bacteria modify the host physiology by altering neurochemical transmission, altering the immune response, and producing metabolites, as van Son et al. (2021) contend.

Metabolic inefficiency, augmented intestinal permeability, and systemic inflammation are all pointers of dysbiosis, an imbalance of the gut microbiota composition, Torres‐Fuentes et al. (2017) declare. Moreover, nutritional and microbial signals can modulate the vagus nerve, a key messenger between the gut and brain. As noted by Longo et al. (2023), it transmits signals from the gut to areas of the brain that regulate hunger and mood. Significant is the vagus nerve and blood–brain barrier being affected by microbial metabolites such as bile acids, neurotransmitter precursors (e.g., tryptophan), and short‐chain fatty acids (SCFAs) regarding satiety and mood (Song et al. 2022; Bliss and Whiteside 2018).

The gut‐brain axis plays a role in obesity that extends beyond merely modulating metabolism; it also influences mental health. Obesity often accompanies mood disorders such as depression and anxiety, which are brought about by chronic inflammation and disrupted gut signaling (Agustí et al. 2018). Thus, the GBA offers a holistic model for exploring the interplay between obesity and mental well‐being. There is a close correlation between obesity and metabolic health, typically defined by factors such as insulin sensitivity, lipid profiles, inflammatory markers, and blood glucose levels (Li, Li, et al. 2025). “Metabolically healthy obesity” was conceived because, much to the contrary, not all individuals who are overweight are metabolically unhealthy. The reality that such individuals still experience greater long‐term health threats, as noted by Gregg and Shaw (2017), suggests that surplus fat is an etiologic change driver in itself. When it comes to managing metabolic health, the gut microbiota reigns supreme. Metabolic disorders linked with obesity may be induced or prevented by alterations in the microbiome, which affect dietary energy extraction, insulin sensitivity, and systemic inflammation (Asadi et al. 2022).

A microbiome profile associated with metabolic outcomes has been proposed by the finding that certain microbial taxa are more prevalent in individuals with obesity, while others are more prevalent in lean individuals (Forte et al. 2020). Metabolic dysregulation is self‐sustained by conditions including leptin and insulin resistance that are compounded by changes in the gut‐brain axis due to obesity, in turn influencing hypothalamic regulation of energy homeostasis (Wu et al. 2019). Another variable complicating the metabolic scenario is gut dysbiosis that results in low‐grade chronic inflammation, a widespread condition observed with obesity (Solas et al. 2017).

The gut microbiota composition and function is essentially regulated by diet. Based on Barber et al. (2023) and García‐Montero et al. (2021), microbial diversity and anti‐inflammatory signatures are associated with nutrient‐dense, high‐fiber diets such as the Mediterranean diet, while Western diets rich in fats and sugars induce dysbiosis and systemic inflammation. Intestinal permeability alterations and neuroinflammatory mechanisms triggered by food can have effects on metabolic and mental health (Gan et al. 2024). Diet, gut microbiome, and mental health have all been correlated in a number of studies. An unhealthy diet, a lack of balance of gut microbes, and depression symptoms were all associated in one study (Taylor et al. 2020).

Madison and Kiecolt‐Glaser (2019) propose that unhealthy diets fuel inflammatory responses and stress‐induced dysbiosis, both of which are bad for psychological health. Zhou and Foster (2015) expose that psychobiotics, or the amalgamation of prebiotics and probiotics, can efficiently treat mental circumstances by leveraging the microbiota‐gut‐brain axis. Such treatments serve to decrease depressive and anxiety symptoms through the establishment of a balance to microbes as well as boosted production of neuroactive chemicals such as serotonin and gamma‐aminobutyric acid (GABA). New studies designate that dietary interventions have potential for utility in obesity‐related mental health prevention as well as for the treatment of such disorders when combined with other therapies.

Based on Merlo et al. (2024), the consequences of treatment in the metabolic and mental spaces might be enhanced using individualized diet plans considering each person's unique microbial profile. A multifaceted network of communication involving metabolic and neuropsychological events, the gut‐brain axis bridges the thin relationship between food patterns, obesity, gut flora, and mental well‐being. The prevention of the global obesity pandemic needs to be achieved by an interdisciplinary, multimodal approach due to the multiple determinants of excess calories among others. The advancement of holistic solutions to enhance metabolic well‐being and mental health in individuals affected by obesity rests on our capacity to comprehend how nutrition affects the GBA.

### 1.1 Aim of the Review

With a focus on how eating habits influence gut microbiota, psychological well‐being, and metabolic well‐being, this review aims to integrate the current understanding of the gut‐brain axis and its role in obesity. Intricate interactions between food, gut bacteria composition, and cognitive factors render obesity a multifactorial disease. Increasing evidence indicates the disruption of metabolic and neurological pathways caused by gut dysbiosis as an etiology of weight gain, insulin resistance, and psychiatric conditions such as anxiety and depression. We contrast the impact of Western diets rich in processed foods, sweets, and saturated fats on gut microbial diversity and inflammation with Mediterranean or plant‐based diets high in fiber, polyphenols, and probiotics. We also examine how emotional eating and chronic stress are psychological factors contributing to obesity through modifying gut‐brain interaction.

The molecular associations between metabolic illnesses, such as insulin resistance and T2D, and gut dysbiosis, are a significant area of research. Therapeutic diets that can influence metabolic and psychological outcomes, such as prebiotics, probiotics, and fiber‐rich nutrition, are examined. Individualized nutrition through microbiome profiling and integrative treatment models that combine dietary intervention, psychological treatment, and medical management are among the future directions covered in this paper. This review discusses the gut‐brain axis as a viable approach to obesity management by integrating the disciplines of psychology, microbiology, and nutrition science.

## 2 Dietary Patterns and Gut Microbiota

Host health is most affected by changing dietary habits; meanwhile, these impact the composition and activity of the gut microbiota. While nutrients play a part in gut flora, dietary habits over time also play a role. Gut dysbiosis, characterized by reduced microbial diversity and elevated levels of pro‐inflammatory bacterial communities, has been linked to a diet ironic in saturated fats, refined carbohydrates, and ultra‐processed foods, as recent evidence suggests (Clemente‐Suárez et al. 2023; Shi 2019).

As per Rizzello et al. (2019), the dysbiotic state is linked with a variety of chronic illnesses, such as obesity, metabolic syndrome, cardiovascular disease, and inflammatory bowel disease. Contrary to this, studies by Barber et al. (2023) and Merra et al. (2020) designate that a Mediterranean diet which is high in fruits, legumes, nuts, olive oil, and moderate wine intake can be supportive of microbial diversity in the gut and even beneficial. Individuals who adhere to this diet to the minute have an increased incidence of bacteria that are SCFA producers, as exposed by an investigation done by De Filippis et al. (2016) and Gundogdu and Nalbantoglu (2023).

Both inflammation and metabolic well‐being are aided by SCFA‐producing bacteria. The diet of an individual can affect his or her immune system responses via the mediation of the interaction among his or her gut bacteria and diet. Two mechanisms whereby the Western diet impacts the immune system are raised systemic inflammation and compromised gut barrier integrity, according to García‐Montero et al. (2021). Contrariwise, foods in the Mediterranean diet have an impact on the microbiota. These foods prevent the immune system from getting out of control, and this decreases the risk of autoimmune and inflammatory diseases (Nagpal et al. 2019; Picchianti Diamanti et al. 2020).

The role of food habits for overall physiological well‐being as well as the balance of gut microbiota is underscored by this interface(Wang et al. 2025). The gut microbiota is recognized by Bremner et al. (2020) as a central mediator of the connection among diet, stress, and psychological health. The usage of microbiota‐friendly dietary practices, such as the Mediterranean diet, is one potential means to augment health and prevent disease via gut control.

### 2.1 Impact of Western Diets vs. Mediterranean Diets on Gut Health

The contemporary Western diet has been interconnected with frequent adverse modifications of the gut microbial community in response to high saturated fat, refined carbohydrate, and fiber intake (Zeng et al. 2024). Such diets have been connected to metabolic and inflammatory disturbances by scientific studies, comprising reductions in microbial diversity and a rise in pro‐inflammatory microbial groups (Rizzello et al. 2019; García‐Montero et al. 2021).

An upsurge in intestinal permeability and systemic inflammation can result in obesity, T2D, and cardiovascular disease (Shi 2019). Contrariwise, the Mediterranean diet, which is characterized by a heavy consumption of beans, nuts, vegetables, whole grains, and olive oil, is recognized to enhance intestinal health. This diet supports varied and balanced microbiota by promoting the dominance of beneficial bacteria such as *Bifidobacterium* and *Lactobacillus* and decreasing the dominance of harmful pathogens, as found in studies (Merra et al. 2020; Barber et al. 2023).

**FIGURE 1:**
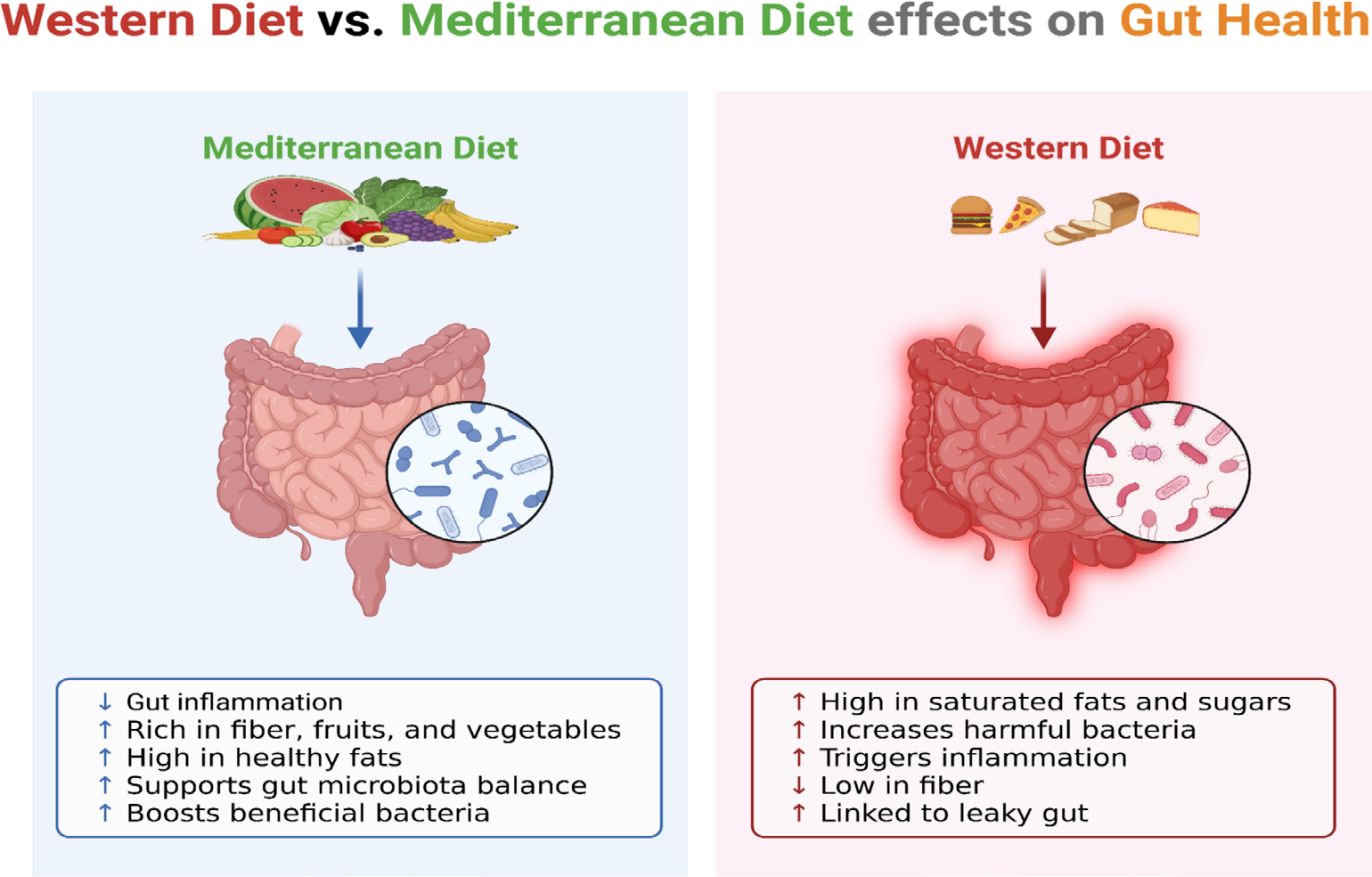
Western diets versus Mediterranean diets on gut health.

The fiber and polyphenol content in this diet supports microbial fermentation. This leads to the production of butyrate and other derivatives of saturated fatty acids. These short‐chain fatty acids (SCFAs) are anti‐inflammatory in nature and function to uphold the intestinal barrier in integrity (Figure 1) (De Filippis et al. 2016). Metabolomics and metagenomics investigations have designated that the gut microbiota is affected by the Mediterranean diet in a manner that supports metabolic and immunological well‐being (Barber et al. 2021; Rinott et al. 2022). Figure 1 shows the western diets vs. Mediterranean diets on gut health.

The gut‐brain axis is linked to stress and emotional control; this has implications for mental health as well, as indicated by research (Bremner et al. 2020). Chicco et al. (2021) and Picchianti Diamanti et al. (2020) resolute that inflammatory bowel diseases (IBD) and rheumatoid arthritis (RA) patients had fewer symptoms when they stuck to the Mediterranean diet exactly. This suggests that the diet possesses anti‐inflammatory and microbiota‐modulating properties. Another element that safeguards against oxidative stress and chronic illness is the significant quantity of polyphenols, vitamins C and E, and other antioxidants consumed (Gantenbein and Kanaka‐Gantenbein 2021).

Microbiota prejudiced by the Western diet is not just incomplete for the immune system. Dysbiosis associated with this diet, unlike the Mediterranean diet that improves immune control by changing the pattern of microbes, impedes immunological functions and promotes an inflammatory environment (García‐Montero et al. 2021). Intervention investigation has recognized that shifting from a Western diet to a Mediterranean diet substantially alters gut microbial patterns. What this suggests is that altering one's diet can improve one's microbiota and keep it healthy (Nagpal et al. 2019; Garcia‐Mantrana et al. 2018).

## 3 Role of Prebiotics, Probiotics, and Fiber in Modulating Gut Microbiota

The development of the gut microbiome is influenced by prebiotics, which are undigested food ingredients that aid in the growth of good bacteria. Certain dietary fibers like inulin, fructooligosaccharides (FOS), and galactooligosaccharides (GOS) might improve the production of SCFA and encourage microbial diversity, as stated by Xu et al. (2022).

Short‐chain fatty acids like butyrate, acetate, and propionate enhance the intestinal barrier's function and possess systemic anti‐inflammatory properties. Beyond the prebiotic effect, probiotics directly enhance populations of useful microbes; these live microorganisms confer health benefits when administered in adequate ratios. Due to their roles in gut health improvement, regulation of immunological function, and prevention of GI infections, *Lactobacillus* and *Bifidobacterium* species are among the most investigated probiotics (Table 1) (He and Shi 2017; Liu et al. 2022).

**TABLE 1:** Comparative analysis of Western versus Mediterranean diets on gut health and microbiota modulation.

Aspect	Western diet impact	Mediterranean diet impact	Role of prebiotics/probiotics/fiber	References
Microbial diversity	Reduced diversity; promotes dysbiosis	Enhances microbial diversity	Prebiotics increase beneficial bacteria such as *Bifidobacteria* and *Lactobacillus*	García‐Montero et al. (2021)
Inflammation	Induces chronic low‐grade inflammation	Anti‐inflammatory through polyphenols and omega‐3	Probiotics modulate immune response and reduce gut inflammation	Clemente‐Suárez et al. (2023)
Gut permeability	Increases gut barrier dysfunction (“leaky gut”)	Preserves gut barrier integrity	Soluble fiber supports mucus production and gut lining integrity	Rizzello et al. (2019)
Short‐chain fatty acid (SCFA) production	Reduced SCFA synthesis due to low fiber intake	Increases SCFA levels, especially butyrate	Prebiotics promote SCFA production, crucial for colonocyte health and anti‐inflammatory effects	Xu et al. (2022)
Obesity/metabolic risk	Promotes insulin resistance and obesity	Protective against metabolic syndrome	Dietary fibers reduce visceral fat and improve insulin sensitivity	Dahiya et al. (2017)
Microbiome stability	Leads to unstable microbiome	Increases resilience and microbial homeostasis	Fiber intake fosters stability and adaptation of beneficial microbes	Beam et al. (2021)
Disease prevention	Linked to higher risk of IBD, diabetes, CVD	Reduces risk of chronic disease via gut modulation	Probiotics restore microbial balance and are linked to reduced risk of certain diseases	Chang et al. (2025); Oniszczuk et al. (2021)
Polyphenol intake	Typically low in processed foods	Rich in polyphenols, which support gut microbial health	Polyphenols act as prebiotics and reduce oxidative stress	Barber et al. (2023)
Mood and brain–gut axis	May negatively influence mental health via dysbiosis	Supports mental well‐being via gut‐brain interaction	Probiotics have potential to improve mood and cognitive function	Bremner et al. (2020)
Lactobacillus/Bifidobacteria	Often decreased	Typically increased	Probiotics boost these beneficial strains	Merra et al. (2020)
Immune modulation	Promotes immune dysregulation	Supports immune tolerance and anti‐inflammatory cytokine production	Prebiotics improve mucosal immunity	Liu et al. (2022)
Oxidative stress	Increases oxidative stress	Rich in antioxidants, lowering oxidative load	SCFAs from fiber reduce oxidative stress	Gantenbein and Kanaka‐Gantenbein (2021)
Microbial fermentation	Limited fermentation substrates	Encourages microbial fermentation via fiber and polyphenols	Fiber fermentation enhances gut health	Holscher (2017)
Cardiometabolic protection	Promotes dysbiosis and atherogenesis	Supports lipid metabolism and glucose regulation	Probiotics help manage blood lipids	Rivera‐Piza and Lee (2020)
Microbial richness	Reduced microbial richness	Associated with greater richness	Diverse fiber sources enhance microbial variety	Mitsou et al. (2017)
RA and autoimmune disease	May worsen autoimmune profiles	May ameliorate autoimmune symptoms via microbiota	Gut modulation can help reduce RA symptoms	Picchianti Diamanti et al. (2020)
Green‐Mediterranean diet	Not applicable	Enhances microbiota diversity and cardiometabolic health	Amplifies fiber and polyphenol intake	Rinott et al. (2022)
Youth diet and microbiota	Often high in sugars/fats, limiting microbial maturity	Encourages healthy microbiome development	Fiber and probiotics improve early‐life microbial profiles	Garcia‐Mantrana et al. (2018)
Fermentation metabolites	Limited due to low fiber and plant intake	Abundant production of bioactive fermentation products	Linked to improved metabolic and immune functions	De Filippis et al. (2016)
Functional probiotics	Rarely used	Often integrated (e.g., yogurt, kefir)	Functional probiotics can enhance host health when consumed consistently	Fei et al. (2023)

The action of western and mediterranean diets on digestive health and modulation of microbiota is compared in Table 1. Synbiotics, being a blend of prebiotics and probiotics, can potentially enhance the function and composition of the microbiota more than either of these individually. Through the restoration of eubiosis and the strengthening of metabolic balance, these blends have shown promise in the treatment of inflammatory disorders, metabolic syndrome, obesity, and metabolic syndrome (Delgado and Tamashiro 2018; Cerdó et al. 2019). Emphasizing the need for specific dietary approaches to change the microbiota for particular health results, recent research proposes categorizing fibers based on their prebiotic potential (Rezende et al. 2021).

Precision nutrition therapies specific to an individual's personalized microbiome and health goals become possible through this approach. An equilibrated microbiota is associated with better metabolic and cardiovascular well‐being, a healthy gut, as well as other systemic compensations of a prebiotic, high‐fiber diet (Li, Zhang, et al. 2025). High fiber enhances insulin sensitivity and decreases LDL cholesterol through a microbial intermediate, based on studies referenced by Oniszczuk et al. (2021) as well as Rivera‐Piza and Lee (2020). Combining second‐generation probiotics, which involve bacterial strains established or naturally set to have augmented functional characteristics, with precise prebiotics could assist individuals suffering from extreme dysbiosis, as per investigation (Fei et al. 2023).

Dietary fibers mark the endocrine and immune systems and ease microbial fermentation, among other activities. Thus, the physiology of the host, microbiota, and nutrition all appear to interrelate in complex manners (Kumar et al. 2020; Ye et al. 2022). Thus, endorsing the intake of fibers, prebiotics, and probiotics ingredients of healthy gut flora is one operative method for sustaining overall health. These consequences contribute to the expanding indication base representing how diet influences the microbiome and how the quality of food influences disease risk and health.

## 4 Psychological Factors in Obesity

Emotional eating and stress behaviors are two means through which psychological fundamentals are intricate in triggering and perpetuating obesity. According to various studies (Van Strien 2018; Devonport et al. 2019), individuals who consume food when sad, anxious, or stressed consume more calories and energy‐dense food. Individuals will consume comfort foods high in fat and sugar when stressed to a high level; for instance, during the COVID‐19 pandemic (Cheng and Wong 2021; Shen et al. 2020).

**FIGURE 2:**
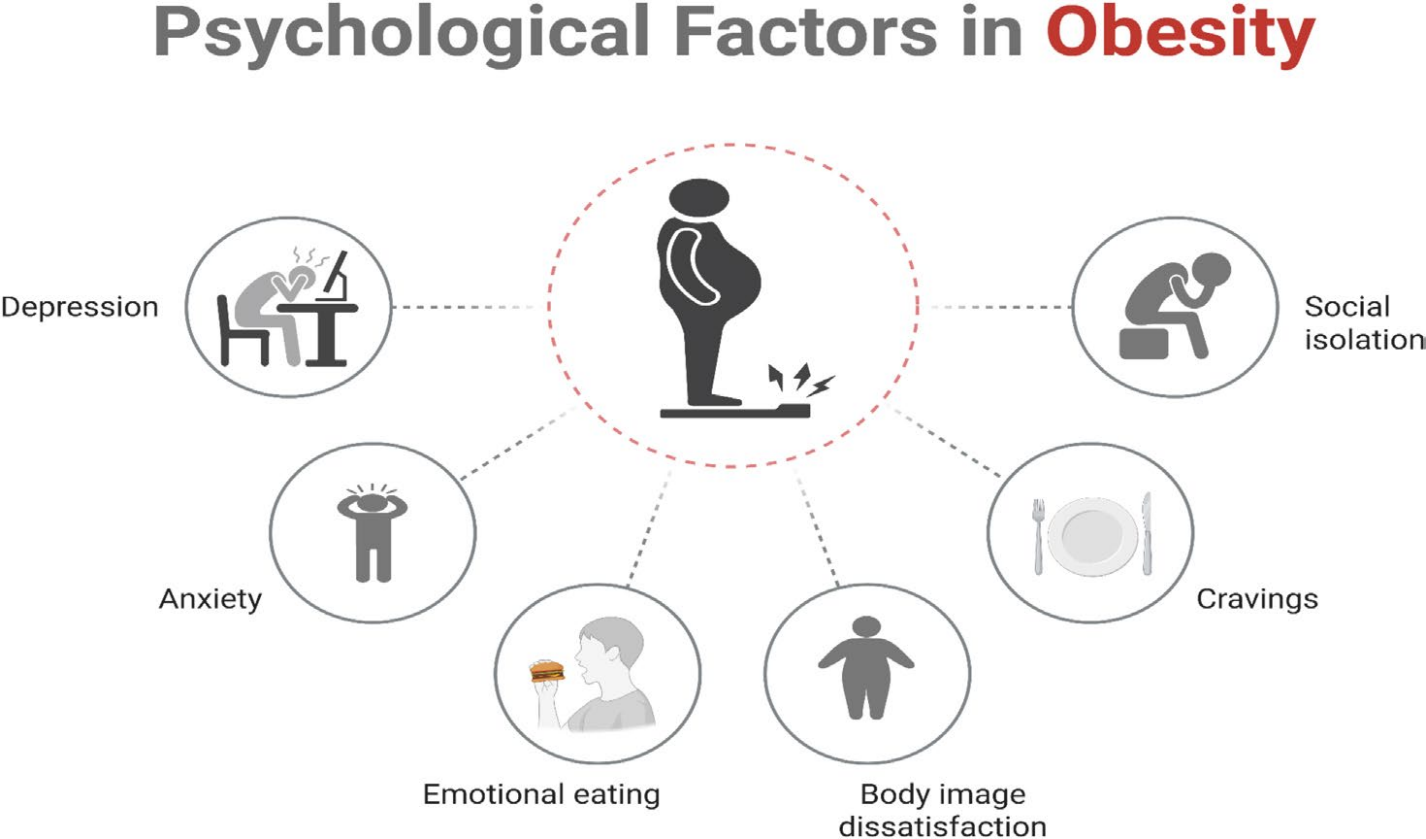
Psychological factors in obesity.

Individuals who struggle with managing their emotions or are psychologically vulnerable are likely to resort to this unhealthy coping strategy even more frequently because they do not know how to manage their emotions in a healthy manner (Figure 2) (Frayn et al. 2018; Trigueros et al. 2020). Obesity and mental disorders such as anxiety and depression have a high correlation with each other. Obese individuals tend to experience mood disorders, and individuals who experience anxiety or depression tend to gain weight, which is partly brought about by emotional eating and inactivity (Fulton et al. 2022; Dakanalis et al. 2023). Figure 2 shows the psychological factors in obesity.

Weight management may be further addressed through the vicious cycle that can be perpetuated by the intricate relationship between psychological discomfort, obesity, and mental health issues (Table 2) (Konttinen 2020; Sharafi et al. 2020). The inclusion of psychological support in the prevention and treatment of obesity programs is essential, as these results identify the importance of mental health in achieving long‐term weight loss objectives. Emotional eating, stress, and mental health connections are a few of the psychological aspects leading to obesity, as indicated in Table 2.

**TABLE 2:** The impact of psychological factors on obesity: Emotional eating, stress, and mental health associations.

Factor/study	Study summary	References	Further details	Key findings
Stress and emotional eating	Stress, emotional eating, and food choices during COVID‐19 in university students	Cheng and Wong (2021)	Impact on emotional eating in university students during COVID‐19	Stress and emotional eating behaviors were heightened during the COVID‐19 pandemic
Stress and emotional eating among university students	Emotional eating and food choices during the pandemic, influenced by perceived stress	Shen et al. (2020)	COVID‐19's emotional eating effects explained in context of stress	Stress during the pandemic increased emotional eating tendencies in students
Emotional eating and food choices during the pandemic	Emotional eating and dietary patterns in individuals with abdominal obesity	Betancourt‐Núñez et al. (2022)	Link between emotional eating and food choices in abdominal obesity	Emotional eating led to unhealthy food choices, particularly in individuals with abdominal obesity
Emotional eating and obesity	Stress exposure, food intake, and emotional state	Ulrich‐Lai et al. (2015)	Impact of stress exposure on emotional states and food intake patterns	Stress contributed to increased emotional eating, exacerbating obesity
Stress exposure and emotional state	Perceived stress and its moderating role in dietary choices and emotional eating	Errisuriz et al. (2016)	Findings on moderating effect of stress management on eating behaviors	Stress management techniques may reduce emotional eating behaviors
Perceived stress and dietary choices	Review of emotions and eating behavior in normal and overweight populations	Devonport et al. (2019)	Role of emotions in determining eating patterns in overweight populations	Emotional factors significantly impact food choices, especially in overweight individuals
Emotions and eating behavior review	Emotional eating prevalence among young Saudi women during COVID‐19	Al‐Musharaf (2020)	Prevalence of emotional eating and its impacts during the pandemic	Emotional eating behaviors were prevalent during the pandemic among young Saudi women
Emotional eating in young Saudi women	Stress and eating behaviors in healthy adults; systematic review and meta‐analysis	Hill et al. (2022)	Effects of stress on eating behaviors as revealed in meta‐analysis	Stress was a major factor influencing eating behaviors in healthy adults
Stress and eating behaviors in adults	Causes of emotional eating and matched treatment for obesity	Van Strien (2018)	Links causes of emotional eating to matching obesity treatments	Identifying emotional eating causes aids in selecting appropriate treatments for obesity
Causes of emotional eating and obesity	Emotional eating and obesity in adults, considering the role of depression, sleep, and genetics	Konttinen (2020)	Factors like sleep and genetics contributing to emotional eating	Depression and poor sleep quality are major contributors to emotional eating in adults
Emotional eating and weight regulation	A qualitative study of compensatory behaviors and concerns related to emotional eating	Frayn et al. (2018)	Compensatory behaviors and concerns regarding emotional eating	Compensatory behaviors, like overeating or restricting food, were linked to emotional eating
Emotional eating and weight regulation	The influence of emotional intelligence on resilience, test anxiety, academic stress, and the Mediterranean diet	Trigueros et al. (2020)	Impact of emotional intelligence on eating behavior and resilience	Emotional intelligence was a protective factor against emotional eating during stressful situations
Influence of emotional intelligence on resilience	Personality, eating styles, and food choices: Direct and indirect effects	Keller and Siegrist (2015)	Personality influencing food choices and eating behaviors	Certain personality traits impact eating styles, which can exacerbate obesity and emotional eating
Personality, eating styles, and food choices	Relationship between obesity, depression, and emotional eating in young adults	Lazarevich et al. (2016)	Link between depression, emotional eating, and obesity development	Depression was strongly linked to emotional eating behaviors in obese young adults
Obesity, depression, and emotional eating	Perceived stress, unhealthy eating behaviors, and severe obesity in low‐income women	Richardson et al. (2015)	Impact of stress on unhealthy eating behaviors in low‐income populations	Stress contributed to unhealthy eating habits and worsened obesity in low‐income women
Perceived stress, unhealthy eating, and obesity	Psychological perspectives on mood and food, the relationship between emotional eating and food‐related emotions	Köster and Mojet (2015)	Emotional responses to food choices and their role in eating behaviors	Mood swings and emotional stress were found to increase food cravings and unhealthy eating
Mood, food, and emotional measurement	The association between obesity and depression/anxiety, particularly in relation to emotional eating	Fulton et al. (2022)	Study on the association of emotional eating with obesity and mental health	Emotional eating was a mediator between obesity and mental health issues like depression and anxiety
Obesity, depression, and anxiety prevalence	Prevalence of anxiety and depression in patients with overweight and obesity, emphasizing emotional eating	Sharafi et al. (2020)	Obesity's relationship with depression and anxiety prevalence	Anxiety and depression were prevalent among those with obesity, exacerbating emotional eating
Prevalence of anxiety and depression in overweight and obese patients	Depression, anxiety, and their role in the development of obesity from childhood to young adulthood	Fan et al. (2024)	Emotional eating's role in exacerbating obesity and mental health issues	Emotional eating played a key role in the development of obesity and mental health disorders
The link between obesity, anxiety, and depression	Impact of depression, anxiety, and obesity development over time, focusing on emotional eating	Adams and Murcia (2016)	Examining depression, anxiety, and obesity development over time	Emotional eating exacerbates both obesity and anxiety, creating a cyclical pattern in patients

### 4.1 Stress, Emotional Eating, and Their Impact on Dietary Choices

**FIGURE 3:**
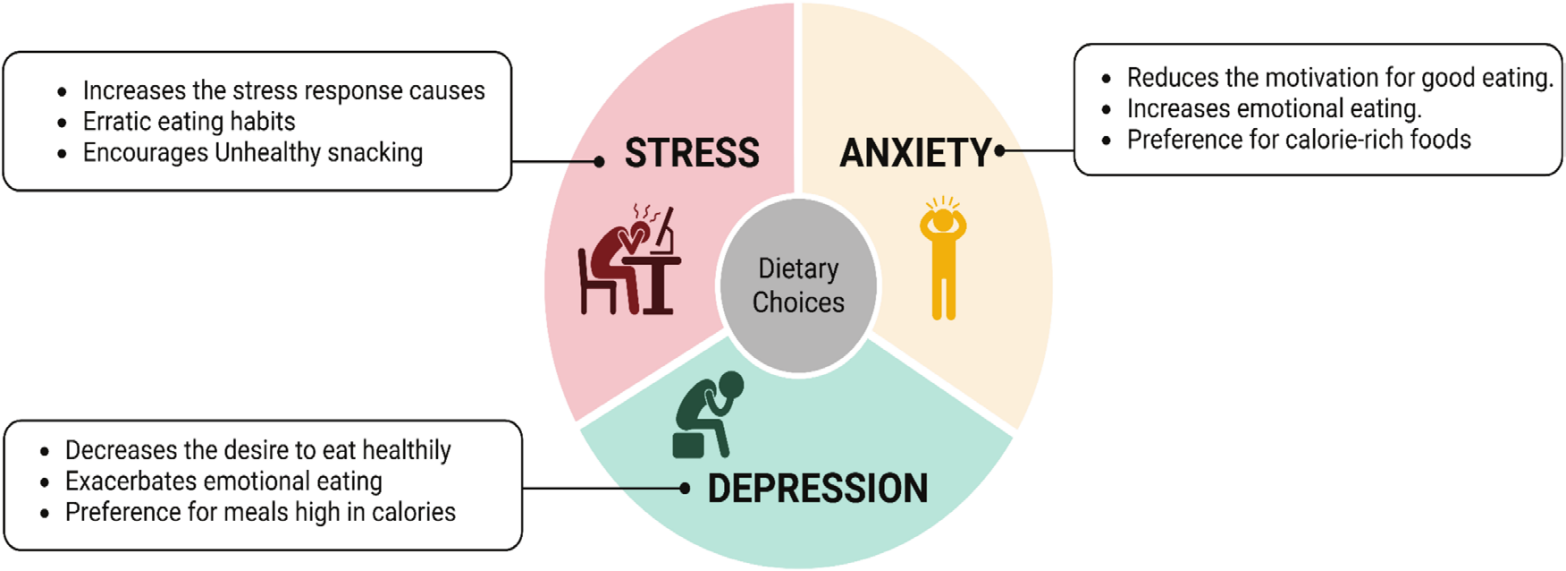
Stress, anxiety, depression and their impact on dietary choices.

Emotional eating and other poor eating habits may be caused by stress, a strong psychological factor that influences dietary habits. When individuals succumb to their emotions instead of their physical hunger, they are emotionally eating (Van Strien 2018). This involves consuming large amounts of calorically dense and palatable food. The stress hormone cortisol increases blood glucose, which in turn elevates hunger and the need for high‐calorie, short‐term comfort foods (Figure 3) (Ulrich‐Lai et al. 2015). Figure 3 shows the stress, anxiety, depression, and their impact on dietary choices.

A cycle of overeating and stress can form when stressors are long‐lasting and constant. Fears generated by the COVID‐19 pandemic could have led to emotional eating and unhealthy food choices among college students (Cheng and Wong 2021). Similarly, Shen et al. (2020) highlighted the point that how individuals view stress has an influence on their food motivation, with many resorting to comfort food in order to cope. Such eating is strongly related to means of coping with and comforting oneself when emotionally upset; it is not merely about indulging. As per various studies, emotional eating is one of the primary reasons behind people's unhealthy eating habits, particularly those who have abdominal fat.

As per Betancourt‐Núñez et al. (2022), overweight individuals in the abdominal area had greater emotional eating scores and their eating patterns were characterized by excessive sugar and fat consumption. That there is a two‐way interaction between one's mental health and his eating habits is proven here. The inability to properly cope with stress has also been linked with emotional eating. Individuals lacking stress management ability are more likely to indulge in bad eating habits, which are likely to result in obesity and heart conditions in the long term (Errisuriz et al. 2016).

Unhealthy eating appears to be motivated, in significant part, by stressed individuals' inability to manage their emotional reactions in ways that bear little relation to food. Based on a systematic review conducted by Devonport et al. (2019), normal‐weight and overweight individuals also suffer from emotional eating, yet based on weight status, the triggers and consequences of this action differ. Overweight persons are more likely to feel guilty, which contributes to stress eating. Aside from worsening mental health and body image concerns, this emotional response can trigger subsequent illustrations of emotional eating. The amount to which these behaviors are organized is significantly affected by emotional intelligence (EI).

Students' resilience, test anxiety, and compliance with the Mediterranean diet were all positively related to EI, as per a study by Trigueros et al. (2020), which indicates that EI can act as a buffer against eating due to stress. Interferences designed to improve emotional regulation can demonstrate to be a valuable resource in the battle against eating disorders. Personality can influence eating habits when stressed. Investigators Keller and Siegrist exposed that persons who are higher in neuroticism and low in conscientiousness will eat incompetently and indulge in their feelings when hungry (Keller and Siegrist 2015).

Persons with such traits are more likely to use food as a crutch throughout times of stress. Further, social and cultural determinants impact the way the bodies of people respond to stress via food. Emotional eating was predominant in young Saudi women during the outbreak (Al‐Musharaf 2020). The same was associated with lifestyle adjustments, disturbance of routine, and increased psychological discomfort. Drawing from these results, it is evident that therapies need to be population‐based with consideration of respective cultural food settings and stresses facing each group. Lastly, there is a robust association between emotional eating and stress, which influences food options in a way that often leads to better health threats and lower quality diet. Stress management, emotional aptitude, and personality characteristics should be the primary areas of successful rehabilitation to help individuals create more enduring eating habits when they feel emotionally upset.

### 4.2 Role of Depression and Anxiety in Obesity Development

There is increasing indication that obesity is made worse by mental health issues such as depression and anxiety, which in turn affect mental health. Emotional eating has been recognized by Frayn et al. (2018) and Van Strien (2018) as a critical factor in the intricate interconnection between mood disorders and weight gain. When individuals with depression or anxiety experience variations in appetite, reduced exercise, and sleep problems, it can result in an imbalance of their energy levels and fat accumulation (Konttinen 2020).

New‐fangled studies have exposed the chemical and neurological mechanisms behind this relationship. Fulton et al. (2022) noted that mood disorder and obesity relate to disruption in neuroendocrine function, that is, hypothalamic–pituitary–adrenal (HPA) axis and sustained low‐grade inflammation. Such biochemical changes favoring insulin resistance and fat accumulation form the substrates for metabolic diseases. There are real concerns regarding young people's risk. Lindberg et al. (2020) discovered that, in comparison to their normal‐weight peers, obese children are significantly more likely to indicate feeling anxious and sad.

Wang et al. (2019) validated this, noting that Chinese children who were overweight had a higher incidence of mood issues, suggesting a potential link between early‐onset obesity and emotional health. The psychological cost of being obese can exacerbate symptoms of depression, rendering it a vicious cycle. Depression, obesity, and emotional eating are interconnected in young adults, says Lazarevich et al. (2016). Body dissatisfaction and social stigma increase the moods of these individuals, rendering emotional eating a highly probable maladaptive strategy. Several studies have indicated that the health burden is amplified when anxiety, depression, and obesity coexist. Obese individuals who are also depressed or anxious are more likely to consume medical services and pay more for such services, as indicated by Nigatu et al. (2017). This identifies the importance of treatment modalities that coordinate mental and physical health.

One interesting connection between obesity and mental health disorders is emotional eating. Most of the relationship between depressive symptom severity and obesity in adolescents was explained by emotional eating, as found by Fox et al. (2016). This indicates the significance of preventing and treating obesity among mentally vulnerable individuals through programs that emphasize control over emotions, especially for children; the environment at home matters. As reported by Kanellopoulou et al. (2022), parental status of mental health, eating styles, and childrearing styles have a significant relationship with children's weight and depression. Thus, therapies addressing families can possibly address both issues simultaneously. When occurring together in adults, depression and obesity have a debilitating impact on their quality of life. The quality of life with respect to health in individuals with both diseases was significantly worse than in individuals with one of these conditions only, as seen from research conducted by Nigatu et al. (2016).

The relevance of diagnosing and treating co‐existing mental illness in patients with obesity is emphasized by this double burden. A biopsychosocial strategy involving medications that inhibit neuroendocrine and inflammatory cascades, psychotherapy, nutrition therapy, and stress management has been suggested by Ouakinin et al. (2018) to confront this interlocked epidemic. Public health policies encompassing these initiatives may enhance the prevention and management of obesity among vulnerable groups of mental illness risk.

### 4.3 Metabolic Health and Diabetes

There is a close link between the composition and role of the gut microbiota, T2D, and metabolic well‐being. T2D is characterized by insulin resistance, and research has associated gut dysbiosis disruption—of populations of microbes with—the condition (Sharma and Tripathi 2019). By changing the gut microbiota conformation, gram‐negative bacteria may release lipopolysaccharides (LPS) into the blood, which can disrupt the intestinal barrier (Di Lorenzo et al. 2019). According to Chen et al. (2021) and Sircana et al. (2018), this may cause chronic inflammation, which in turn constrains insulin signaling. Furthermore, dysbiotic changes enhance beta‐cell injury and glucose intolerance by triggering oxidative stress and mitochondrial dysfunction. As distinguished by Tangvarasittichai (2015) and Petrick et al. (2020), this further supports the metabolic abnormalities in T2D.

Apart from factors associated with the digestive system, lifestyle habits and diet also have significant roles in upholding metabolic health. Based on studies (Wen and Duffy 2017; Zhang et al. 2021), a healthy fat, fiber, and antioxidant‐rich diet, such as the Mediterranean diet, can induce an enhanced gut microbiota composition, which in turn augments insulin sensitivity and glycemic control. Wang, Lu, et al. (2020) exposed that fecal microbiota transplantation (FMT) and other interferences reverse insulin resistance, showing the therapeutic value of targeting the gut microbiota. The results of this study designate that augmenting gut health using dietary and microbiological interventions may be a means to prevent or manage T2D.

### 4.4 The Significance of the Intestinal Barrier in Obesity and Gut‐Brain Axis Dysfunction

A vital defense mechanism, the intestinal barrier prevents bacteria and their products, such as lipopolysaccharides (LPS), from crossing into the bloodstream (Ghosh et al. 2020). It consists of epithelial cells, mucus, and tight junction proteins and is crucial to keeping the gut in‐house (Capaldo et al. 2017). Metabolic endotoxemia and low‐grade inflammation are linked with obesity, resulting in insulin resistance and systemic inflammation as a result of enhanced intestinal permeability, referred to at times as “leaky gut” (Ghosh et al. 2020). The mucus layer is the first line of defense against infections, comprised of mucins secreted by goblet cells (Vancamelbeke and Vermeire 2017).

Obesity aggravates metabolic derangement because of the modification of tight junction proteins, which control paracellular permeability (Sánchez de Medina et al. 2014). There is evidence that a diet rich in saturated fats and refined carbohydrates could impact the expression of tight junction proteins and reduce mucus thickness, both reducing the integrity of the intestinal barrier (Lopetuso et al. 2015). As described by Shu et al. (2023), psychological disorders and neuroinflammation may be caused through the gut‐brain axis by bacterial LPS entering circulation, triggering toll‐like receptors (TLRs), and initiating pro‐inflammatory cytokine production. Conversely, fiber‐rich dietary interventions can enhance barrier function through enhanced mucus secretion and tight junction integrity through the production of short‐chain fatty acids (SCFA) (Capaldo et al. 2017). It is, thus, important to understand the integrity of the intestinal barrier in order to realize the two‐way relationship between metabolic and psychological well‐being in obesity.

## 5 Gut Dysbiosis and Insulin Resistance in T2D

The human gut microbiota controls host metabolism, immunological functions, and inflammation; it is closely linked to the pathophysiology of T2D (Yin et al. 2025). Recent scientific debates have focused on dysbiosis, a disruption in the balance of gut bacteria, as a potential etiology for insulin resistance (Sharma and Tripathi 2019). Alterations in microbial diversity, particularly decreases in symbiotic bacteria such as *Akkermansia muciniphila* , have been associated with compromised gut barrier function and elevated systemic inflammation (Chen et al. 2021).

Inflammatory variations often impair insulin signaling pathways. Increased lipopolysaccharides (LPS), cell wall components of gram‐negative bacteria, have been correlated with dysbiosis, and metabolic endotoxemia has been revealed to induce insulin resistance (Salguero et al. 2019). Further proof of the connotation of dysbiosis with T2D was exposed using experiments on mouse models that had beforehand received translocation of gut microbiota from diabetic individuals (Liaqat et al. 2021). Additionally, oxidative stress has been found to be linked with gut dysbiosis, which further endorses insulin resistance and beta‐cell dysfunction (Tangvarasittichai 2015).

A feedback loop is established by this oxidative environment, which further disrupts the microbiome and sustains metabolic dysfunctions. These explanations propose a possible physiological connection among microbial composition and insulin resistance. The gut‐liver axis, or interaction among the two organs, is another etiological factor for this disease. Irrespective of body mass and insulin resistance, nonalcoholic fatty liver disease (NAFLD) that frequently comes with T2D has been linked with dysbiosis, that is, a systemic implication of increased microbial variation (Da Silva et al. 2018; Tanase et al. 2020).

Recent clinical studies in human subjects suggest control of gut microbiota to be highly impactful for clinical trials. For instance, Wang, Yang, et al. (2020) report that fecal microbiota transplantation (FMT) from lean donors may reverse the insulin resistance and islet function of patients with metabolic syndrome. However, the safety and efficacy of such treatments over the long term remain to be investigated. Combined, the evidence demonstrates that gut dysbiosis is a cause of T2D, not only a symptom of it. It is important to maintain the health of the gut environment because shifts in microbes may influence glucose metabolism (Sikalidis and Maykish 2020).

### 5.1 Dietary Interventions for Metabolic and Psychological Outcomes

Dietary therapies have emerged as non‐pharmacological interventions to improve metabolic and psychological outcomes, considering the relationship between gut microbiota and T2D. Dietary factors significantly impact the gut microbiota, and these, in turn, influence the diversity of microbes, the immune system's functioning, and inflammatory processes (Zhang et al. 2021; Wen and Duffy 2017). There is robust evidence that the high‐fiber, polyphenolic, and omega‐3‐rich Mediterranean diet benefits insulin resistance, mood, and cognitive function.

Parletta et al. (2019) and Jacka et al. (2017) reported that such a dietary lifestyle enhanced metabolic regulation and psychological well‐being by raising gut microbial richness and lowering pro‐inflammatory taxa. Randomized controlled trials have demonstrated that whole‐diet therapies, rather than individual nutrient supplementation, are most effective in reducing depressive symptoms and glycemic improvement (Opie et al. 2015; Firth et al. 2019).

Oriach et al. (2016) discovered that the microbiome mediates changes in mental health that are related to nutrition. These benefits are likely due to synergistic effects on the gut‐brain axis. Diet may be therapeutic for mental illness, as suggested by the growing field of nutritional psychiatry that often co‐exists with metabolic disorders. Based on new research, there is promise that plant and anti‐inflammatory diets can reduce the mood and metabolic impairments of overweight individuals (Medawar et al. 2019; Adan et al. 2019). Wholistic improvement may also be had through mindfulness‐based eating habits. These interventions enhance physical and mental health by lowering stress and changing dietary habits (Table 3) (Rogers et al. 2017).

**TABLE 3:** The role of gut dysbiosis in metabolic dysfunction and potential dietary interventions.

Mechanism of gut dysbiosis in metabolic dysfunction	Impact on insulin resistance/diabetes	Dietary interventions	Psychological benefits	References
Reduced microbial diversity	Decreased SCFA production → impaired glucose metabolism	High‐fiber diet (prebiotics)	Reduced depression symptoms	Chen et al. (2021)
Increased firmicutes/bacteroidetes ratio	Enhanced energy harvest → obesity → T2DM risk	Mediterranean diet	Lower anxiety scores	Sircana et al. (2018)
LPS‐producing bacteria (e.g., enterobacteriaceae)	Chronic inflammation → insulin resistance	Probiotic supplementation (Lactobacillus)	Improved stress resilience	Sharma and Tripathi (2019)
Decreased *Akkermansia muciniphila*	Gut barrier dysfunction → metabolic endotoxemia	Polyphenol‐rich foods (berries, nuts)	Better mood regulation	Zhang et al. (2021)
Bile acid metabolism disruption	Altered FXR/TGR5 signaling → glucose intolerance	Omega‐3 fatty acids (fish oil)	Reduced emotional eating	Wang, Lu, et al. 2020
Butyrate deficiency	Mitochondrial dysfunction in hepatocytes	Resistant starch supplementation	Cognitive improvement	Sikalidis and Maykish (2020)
Increased pathobionts (e.g., *E. coli* )	Oxidative stress → β‐cell damage	Fermented foods (kefir, kimchi)	Lower inflammation‐related depression	Chen et al. (2023); Tangvarasittichai (2015)
TMAO‐producing microbiota	Endothelial dysfunction → diabetic complications	Plant‐based diet	Enhanced gut‐brain axis signaling	Tanase et al. (2020)
Gut permeability (“leaky gut”)	Systemic LPS → adipose tissue inflammation	Low‐FODMAP diet (for dysbiosis)	Improved sleep quality	Petrick et al. (2020)
SCFA receptor downregulation	Impaired GLP‐1 secretion → hyperglycemia	Inulin‐type fructans	Anxiety reduction	Wen and Duffy (2017)
Methanogen overgrowth (e.g., Methanobrevibacter)	Slowed intestinal transit → weight gain	Time‐restricted feeding	Stress coping benefits	Da Silva et al. (2018)
Candida overgrowth	Immune activation → insulin resistance	Antifungal herbs (garlic, turmeric)	Fewer food cravings	Slyepchenko et al. (2016)
Virome alterations	Viral infections triggering autoimmunity	Vitamin D supplementation	Reduced depressive episodes	Liaqat et al. (2021)
Histamine‐secreting microbes	Mast cell activation → metabolic inflammation	Low‐histamine diet	Better emotional stability	Firth et al. (2019)
Dopamine‐metabolizing bacteria	Reward system dysfunction → overeating	Tyrosine‐rich foods (eggs, legumes)	Motivation enhancement	Opie et al. (2015)
GABA‐producing microbiota depletion	HPA axis dysregulation → cortisol dysbalance	GABA‐enhancing foods (kimchi, tempeh)	Antidepressant effects	Chu et al. (2019)
Tryptophan metabolism shift	Kynurenine pathway activation → neuroinflammation	Tryptophan sources (turkey, seeds)	Serotonin boost	Parletta et al. (2019)
Zonulin upregulation	Tight junction impairment → endotoxemia	Gluten‐free diet (for sensitive individuals)	Reduced brain fog	Jacka et al. (2017)
B‐vitamin synthesizing microbes deficit	Homocysteine accumulation → vascular damage	Nutritional yeast supplementation	Neurological protection	Vancampfort et al. (2021)
Circadian rhythm‐disrupting microbiota	Melatonin dysregulation → metabolic syndrome	Meal timing synchronization	Improved circadian rhythms	Medawar et al. (2019)

Overweight or obese individuals with metabolic syndrome or T2D can benefit significantly from them. Table 3 presents data on the potential dietary interventions and the role of gut dysbiosis in metabolic dysfunction. Both eating patterns and the makeup of one's microbiota affect one's mental well‐being. A cycle of viciousness can be sustained when individuals with depression and anxiety alter their diets, resulting in increased consumption of unhealthy foods and poorer metabolic dysfunction (Bremner et al. 2020).

In type 2 diabetics, the optimal outcomes might be achieved with the combination of dietary modification and psychological counseling directed to the axis microbiota‐gut‐brain (Marx et al. 2017, 2021). A personalized diet based on the individual composition of every subject's microbiota and his psychological status is the following step in metabolic treatment. Lastly, in the interest of both metabolic well‐being and mental toughness, nutritional therapies are essential. T2D, as well as associated mental distress, might be optimally managed through attention to gut health through diet.

## 6 Conclusion and Future Perspectives

Diet, gut microbiota, and metabolic and psychiatric complications of obesity are all linked by the gut‐brain axis. Fiber‐rich diets and the Mediterranean diet promote mental health and microbial diversity, but Western diets worsen gut dysbiosis, inflammation, and insulin resistance. There is a vicious cycle in the etiology of obesity in which psychological disturbances, including stress and depression, make poor dietary choices even worse. Metabolic well‐being and psychological functioning can be enhanced by dietary treatments aimed at gut flora, such as prebiotics and probiotics. An effective approach to the treatment of obesity has to be multidisciplinary in outlook, encompassing food habits, gut health, and mental well‐being. In a bid to break the vicious cycle of emotional eating and metabolic impairment, the evidence suggests that nutritional treatments have to be coupled with psychosocial treatments. The long‐term effects of diet on the gut microbiota and mental health of obese individuals should be the focus of future research.

The application of gut microbiota profiling to guide individualized dietary advice is a promising new frontier in the battle against obesity. New findings on the microbiome can help unlock personalized dietary advice that supports metabolic well‐being through microbe balance. In addition, precision nutrition methodologies may be enhanced through the integration of microbiome analysis with artificial intelligence. Treatment for obesity can make a sea change if interdisciplinary teams collaborate to enhance patients' physical health, mental health, and diets. Clinical trials need to investigate synbiotics (probiotics with prebiotics) and psychobiotics (psychiatric interventions that act on the microbiota) to validate their effectiveness. With a view to reducing the metabolic and psychological aspects of the global pandemic of obesity, public health promotion campaigns promoting gut‐friendly diets could be effective. New biomarkers for the early prediction of obesity risk may be discovered through increased research into the gut‐brain axis, permitting proactive treatments. Evidence‐based, holistic treatment protocols for obesity will need cooperation between physicians, psychologists, and nutritionists.

## Author Contributions

**Faiyaz Ahmed:** writing – original draft (equal). **Muhammad Tayyab Arshad:** writing – review and editing (equal). **Sammra Maqsood:** data curation (equal). **Ali Ikram:** supervision (equal). **Kodjo Théodore Gnedeka:** validation (equal).

## Ethics Statement

This study did not involve humans or animals.

## Consent

This study did not involve humans.

## Conflicts of Interest

The authors declare no conflicts of interest.

## Data Availability

The data supporting this study's findings are available from the corresponding author upon reasonable request.
